# Characterization of Vancomycin Resistance and Associated Genes in Clinical Isolates of Enterococcus Species

**DOI:** 10.7759/cureus.111791

**Published:** 2026-06-30

**Authors:** Madhumala Shanmugasundaram, MuthuLakshmi BackiaSubramanian, Shanthi Mariappan, Uma Sekar, Kennedy Kumar, Binesh Lal Y, Rhea Michelle J Khodabux, Sribal Selvarajan

**Affiliations:** 1 Microbiology, Sri Ramachandra Institute of Higher Education and Research, Chennai, IND; 2 Clinical Microbiology, Christian Medical College, Chennai, IND

**Keywords:** healthcare, minimum inhibitory concentration, polymerase chain reaction, vana gene, vanc1, vancomycin-resistant enterococci

## Abstract

Introduction

Vancomycin is the drug of choice for multidrug-resistant *Enterococcal* infections. Increased use of vancomycin has resulted in greater minimum inhibitory concentration (MIC) values and resistance. The objective of this study was to detect and characterize vancomycin resistance among clinical *Enterococcus* isolates using phenotypic susceptibility testing and correlation with molecular analysis of vancomycin resistance genes (*vanA*,* vanB*,* vanC1*,* vanC2/3*,* vanD*,* vanE*, and *vanG*).

Materials and methods

A total of 266 clinically significant, consecutive, nonrepetitive *Enterococcus *specieswere identified from clinical isolates collected from 2023 to 2024 in a tertiary care hospital. Sources of the isolates included urine (98), blood (19), and exudative specimens (149). Using the Kirby-Bauer disc diffusion method, antibiotic susceptibility was tested for various antimicrobial agents, including ampicillin, erythromycin, ciprofloxacin, nitrofurantoin, vancomycin, and linezolid. The agar dilution technique was performed as per Clinical and Laboratory Standards Institute (CLSI) 2025 guidelines to ascertain the MIC for vancomycin. Polymerase chain reaction (PCR) was employed to detect the genes encoding vancomycin resistance, namely, *vanA*,* vanB*,* vanC1*,* vanC2/3*,* vanD*,* vanE*,and *vanG.*

Results

Among 266 isolates, vancomycin resistance was detected in 74 isolates with MIC ≥ 32 μg/mL (*Enterococcus faecium *- 57, *Enterococcus faecalis - *9, *Enterococcus gallinarum *- 7, *Enterococcus casseliflavus - *1). All the 74 carried van gene. *vanA *was detected in 70 and *vanC1* in 3 isolates of *E. gallinarum. *One isolate of *E. gallinarum* harbored both *vanA *and *vanC1*. None of the vancomycin-resistant *Enterococcus *(VRE) isolates in this study carried *vanB*,* vanC2/3*,* vanD*,* vanE*, or *vanG*.

Conclusion

The increase in VRE has alarmed the global community. More recently, the emergence of linezolid-resistant *Enterococci* is a cause for concern. Hence, there is an imperative need for in-depth research into the mechanisms behind *VRE*. In this study, the *vanA* genotype is more widely distributed in clinical isolates.

## Introduction

*Enterococci* are Gram-positive, facultative anaerobic cocci arranged in short chains and are important causes of nosocomial infections such as urinary tract infection, soft tissue infections, sepsis, and meningitis. The growing burden of vancomycin-resistant *Enterococci *(VRE) in healthcare represents a serious clinical and public health concern due to multidrug resistance, prolonged hospitalization, increased mortality, and limited treatment options [1]. VRE were initially documented in Europe in 1988, while the first report from India appeared in 1999 in New Delhi [2]. In 2017, the World Health Organization included VRE among the most important antibiotic-resistant bacteria in its Global Priority List [3].

One of the most important features of *Enterococci* is their remarkable ability to resist a wide range of antimicrobial agents through both intrinsic and acquired resistance mechanisms. Among the different species, *Enterococcus faecium* has gained particular attention because of its capacity to accumulate resistance to multiple classes of antibiotics. Since the 1980s, several studies have reported a steady rise in resistance among *E. faecium* to fluoroquinolones, aminoglycosides, and glycopeptides, including vancomycin. In contrast, although *Enterococcus faecalis* often exhibits resistance to aminoglycosides, it generally remains susceptible to ampicillin and vancomycin [4].

Vancomycin is a glycopeptide antibiotic that exerts its antibacterial activity by binding to the D-alanyl-D-alanine terminus of peptidoglycan precursors, thereby interfering with bacterial cell wall synthesis [5]. In *Enterococci*, resistance to vancomycin mainly arises through alterations in this target site. These changes modify the terminal amino acid sequence of the cell wall precursor, resulting in a marked reduction in the binding affinity of glycopeptide antibiotics. Replacement of D-alanyl-D-alanine with D-alanyl-D-lactate leads to an approximately 1,000-fold decrease in vancomycin binding, while substitution with D-alanyl-D-serine results in a smaller, nearly sevenfold reduction [6]. To date, nine vancomycin resistance gene clusters have been described in *Enterococci*. These resistance determinants are generally grouped according to the type of ligase enzyme they encode, which is responsible for the synthesis of the altered cell wall precursors [6,7]. The first group, including *vanA*, *vanB*, *vanD*, and *vanM*, encodes D-alanyl-D-lactate ligase, whereas the second group, comprising *vanC*, *vanE*, *vanG*, *vanL*, and *vanN*, encodes D-alanyl-D-serine ligase. Among these, *vanC* is unique because it mediates intrinsic resistance and is typically found in species such as *Enterococcus gallinarum* and *Enterococcus casseliflavus*. The remaining van gene clusters are primarily associated with acquired glycopeptide resistance [8,9]. Globally,* vanA* and *vanB *remain the most commonly reported determinants of vancomycin resistance and are predominantly identified in *Enterococcus faecium* [10].

Numerous studies indicate that patients with VRE bacteremia exhibit higher mortality rates than those infected with susceptible strains. Hospitalized patients colonized with VRE contribute to the dissemination of these resistant bacteria. Major concerns regarding VRE include limitations in therapeutic options and the potential for horizontal gene transfer of vancomycin resistance to other gram-positive pathogens. Notably, if vancomycin resistance genes are transferred to methicillin-resistant *Staphylococcus aureus* (MRSA), it can give rise to vancomycin-resistant *Staphylococcus aureus*, which is unresponsive to conventional antibiotic therapy [11-13].

Several studies from India have reported the prevalence of VRE and characterized the commonly occurring *vanA* and *vanB *resistance determinants [2,4]. Studies investigating the distribution of less commonly studied van genes, including *vanC1*,* vanC2/3*,* vanD*,* vanE,* and* vanG*, remain scarce. Comprehensive molecular characterization of VRE isolates is important for detecting both common and uncommon resistance determinants and for understanding the diversity of vancomycin resistance mechanisms among clinical *Enterococcus* isolates.

Study objective

The objective of this study was to characterize vancomycin resistance among clinical isolates of *Enterococcus species* using phenotypic methods based on minimum inhibitory concentration (MIC) determination and correlation with genotypic analysis of vancomycin resistance determinants (*vanA*, *vanB*, *vanC1*, *vanC2/3*, *vanD*, *vanE*, and *vanG*).

## Materials and methods

Study settings

The present study was an in vitro, laboratory-based, prospective study conducted at a 1,600-bed tertiary care university teaching hospital. Ethical approval for this study was obtained from the Institutional Ethics Committee (IEC) of Sri Ramachandra Institute of Higher Education and Research (Reference Number: IEC-N1/23/AUG/88/43, dated August 25, 2023).

Clinical isolates and identification

A total of 266 clinically significant, consecutive, and non-duplicate isolates collected during 2023-2024 were included in the study. The isolates were obtained from urine (n = 98), blood (n = 19), and exudative specimens (n = 149). Clinical significance was determined by correlating them with patient history, detecting intracellular organisms on Gram-stained smears, and observing significant growth in culture media. Species-level identification was carried out using conventional biochemical methods and automated techniques, including matrix-assisted laser desorption ionization-time of flight mass spectrometry (MALDI-TOF MS) from bioMérieux Marcy l'Etoile, France.

Antibiotic susceptibility

Disc Diffusion

Antibiotic susceptibility testing was performed by the disc diffusion method following the Clinical and Laboratory Standards Institute (CLSI) 2025 guidelines. The list of antibiotics included ampicillin (10 µg), vancomycin (30 µg), linezolid (30 µg), nitrofurantoin (300 µg; urinary isolates), and ciprofloxacin (5 µg; urinary isolates). Erythromycin (15 µg) was used only for non-urinary isolates. Quality control testing was carried out using *Enterococcus faecalis* ATCC 29212 as the control strain.

Minimum Inhibitory Concentration

The agar dilution method was used for the MIC test, which is used to test vancomycin susceptibility. Agar plates were prepared with 12 different concentrations varying from 0.125 to 128 µg/mL from the stock solution. Three to four isolated *Enterococcus *colonies were inoculated in peptone broth and adjusted to a 0.5 McFarland standard. Inoculation was done on the gridded Mueller-Hinton agar plates using a sterile loop. These plates were then incubated at 37°C for 24 hours. Isolates that showed an MIC value of ≥32 µg/mL were phenotypically resistant to vancomycin. The ATCC reference strain *Enterococcus faecalis *ATCC 29212 was utilized for quality assurance during testing. Results were interpreted according to the CLSI 2025 guidelines.

Molecular analysis

DNA Extraction

A loopful of clinical *Enterococcus *colonies was inoculated into Luria-Bertani broth (HiMedia Laboratories, Mumbai, India) and incubated overnight at 37°C. Following incubation, the bacterial culture was centrifuged at 10,000 rpm for 10 min to obtain a pellet. The pellet was then resuspended in 400 μL of sterile distilled water and subjected to boiling at 100°C for 10 min to facilitate cell lysis. After cooling to room temperature, the lysate was centrifuged again at 10,000 rpm for 10 min. The resulting supernatant containing the extracted DNA was collected and used as the template for subsequent polymerase chain reaction (PCR) [14].

Polymerase Chain Reaction

Detection of vancomycin resistance genes (*vanA*, *vanB*, *vanC1, vanC2/3*, *vanD*, *vanE*, and *vanG*) was performed using PCR for all isolates. The template DNA was extracted by the boiling lysis method. Each PCR reaction was set up in a total volume of 10 µL, consisting of 5 µL of master mix, 1 µL of nuclease-free water, 0.5 µL each of forward and reverse primers for both genes, and 2 µL of template DNA. Two separate duplex PCR were employed for the detection *vanA/vanB *and *vanC1/vanC2/3*, whereas multiplex PCR was used for the detection of *vanD, vanE*, and *vanG*. The amplified products were then visualized under a UV transilluminator [14,15]. Primer sequences and cyclic conditions details are provided in Table 1. The PCR products were resolved on a 2% agarose gel prepared with ethidium bromide. Electrophoresis was carried out in 1X Tris-acetate-EDTA (TAE) buffer at 100 V for 25 minutes. The amplified products were then visualized under a UV transilluminator [14]. PCR products corresponding to each detected van gene were purified and sequenced for confirmation. Strains confirmed by sequencing were then included as positive controls for *vanA *and *vanC1 *in subsequent PCR experiments.* Enterococcus faecalis *ATCC 51299 was used as a positive control for *vanB*,while sterile Milli-Q water was used as the negative control; both were included in each run.

**Table 1:** PCR primer and cycling conditions for the detection of van genes

Gene	Primer	Cycling conditions	Base pair	Ref.
vanA	[F]‑GGGAAAACGACAATTGC [R]‑GTACAATGCGGCCGTTA	Initial denaturation step at 94°C for 2 min, followed by 25 cycles of amplification (94°C for 60 s, 55°C for 60 s, and 72°C for 60 s), and an extension at 72°C for 5 min	732	[15]
vanB	[F]‑ATGGGAAGCCGATAGTC [R]‑GATTTCGTTCCTCGACC		635	[15]
VanC1	[F]‑GGCATCGCACCAACAATGGA [R]‑TCCTCTGCCAGTGCAATCAA	Initial denaturation step at 94°C for 3 min, followed by 30 cycles of 94°C for 1 min, 57°C for 45 s, and 72°C for 1 min. A final extension step was carried out at 72°C for 5 min.	902	[5]
vanC2/3	[F]‑TTCAGCACTAGCGCAATCG [R]‑TCACAAGCACCGACAGTCAA		663	[5]
vanD	[F]‑TGTGGGATGCGATATTCAA [R]‑TGCAGCCAAGTATCCGGTAA	Initial denaturation step at 94°C for 2 min, followed by 25 cycles of amplification (94°C for 60 s, 55°C for 60 s, and 72°C for 60 s), and an extension at 72°C for 5 min.	500	[15]
vanE	[F]‑TGTGGTATCGGAGCTGCAG [R]‑ATAGTTTAGCTGGTAAC		430	[15]
vanG	[F]‑CGGCATCCGCTGTTTTTGA [R]‑GAACGATAGACCAATGCCTT		941	[15]

DNA Sequencing

Representative PCR isolates were analyzed by automated DNA sequencing. The obtained sequences were edited and assembled using BioEdit software version 7.0.5.3 (Ibis Biosciences, Carlsbad, CA). Sequence similarity was assessed using the Basic Local Alignment Search Tool (BLAST) version 2.17.0 available through the NCBI (National Center for Biotechnology Information) database. The sequences were submitted to GenBank, and accession numbers were obtained.

Data Presentation and Software

Data were compiled and analyzed using Microsoft Excel (Microsoft Corp., Redmond, WA). Graphical representations were prepared using Microsoft Word (Microsoft Corp., Redmond, WA).

Statistical analysis

Statistical analysis was performed using GraphPad Prism software version 11.0.2 (GraphPad Software, San Diego, CA). Categorical variables, including VRE isolates and patient admission status (ward/ICU), were analyzed using the chi-square test. A p-value of <0.05 was considered statistically significant.

## Results

A total of 266 *Enterococcus* isolates were included in the present study. The species distribution of *Enterococcus *isolates is shown in Table 2.

**Table 2:** The distribution of Enterococcus species

Species	Total	Percentage
Enterococcus faecalis	128	48.1%
Enterococcus faecium	117	44.0%
Enterococcus gallinarum	7	2.6%
Enterococcus avium	6	2.3%
Enterococcus hirae	4	1.5%
Enterococcus raffinosus	2	0.7%
Enterococcus casseliflavus	1	0.4%
Enterococcus durans	1	0.4%

Antimicrobial susceptibility pattern

The antimicrobial resistance profile of the isolates showed resistance to ampicillin in 57% (152/266), ciprofloxacin in 84.6% (83/98), nitrofurantoin in 52% (51/98), erythromycin in 82.1% (138/168), vancomycin in 27.8% (74/266), and linezolid in 9.4% (25/266) of the isolates.

MIC of VRE species

**Figure 1:**
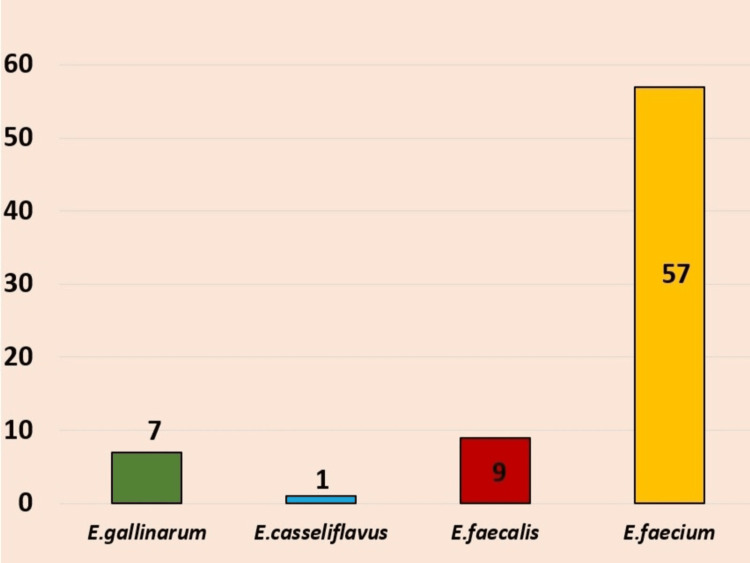
Species distribution of vancomycin-resistant Enterococcus

Out of 266 isolates, 74 showed resistance to vancomycin with MIC ≥ 32 µg/mL. Among the 74 VRE isolates, urine was the most common source (34/74 (45.9%)), followed by exudative specimens (32/74 (43.2%)) and blood (8/74 (10.8%)). MIC results interpreted as per the CLSI guidelines 2025 are depicted in Table 3. Species distribution of VRE is illustrated in Figure 1.

**Table 3:** MIC results were interpreted according to the CLSI guidelines (2025) MIC results for vancomycin were interpreted according to CLSI 2025 breakpoints for *Enterococcus *spp.: susceptible, ≤4 µg/mL; intermediate, 8–16 µg/mL; and resistant, ≥32 µg/mL. Of the 266 isolates tested, 192 (72.2%) were susceptible, and 74 (27.8%) were resistant to vancomycin. None of the isolates exhibited intermediate susceptibility (8–16 µg/mL). CLSI: Clinical and Laboratory Standards Institute; MIC: Minimum inhibitory concentration.

MIC range (µg/mL)	0.125	0.25	0.5	1	2	4	8	16	32	64	128	>128
No. of. isolates	0	1	25	77	60	29	0	0	9	21	17	27

Molecular analysis of vancomycin resistance genes

Among the 266 *Enterococcus isolates*, 74 showed phenotypic resistance to vancomycin (MIC ≥ 32 µg/mL). PCR analysis detected van genes in 84/266 (31.6%) isolates, including all 74 VRE isolates. Of the 84 van gene-positive isolates, 70 carried *vanA*, and 3 carried *vanC1. vanA* and *vanC1 *coexisted in one isolate, while the remaining 10 *vanA*-positive isolates were phenotypically susceptible to vancomycin. None of the isolates carried *vanB*, *vanC2/3*, *vanD*, *vanE*, or *vanG*. Representative PCR amplicons were submitted to GenBank, and accession numbers were obtained (*vanA*: PQ568068 and *vanC1*: PQ568069). Species-wise distribution of van genes is illustrated in Table 4.

**Table 4:** Distribution of van genes among species

Species	*vanA *(n = 70)	*VanC1* (n = 3)	Both *vanA *and *vanC1*
*Enterococcus faecium *(n = 57)	57	0	0
*Enterococcus faecalis *(n = 9)	9	0	0
*Enterococcus gallinarum *(n = 7)	3	3	1
*Enterococcus casseliflavus *(n = 1)	1	0	0

 The association between phenotypic resistance and genotypic resistance is shown in Table 5.

**Table 5:** Association between phenotypic resistance and genotypic resistance Vancomycin MIC distribution among *vanA *and *vanC1*-positive *Enterococcus* isolates. One isolate was positive for both *vanA* and *vanC1* genes and showed a vancomycin MIC of 32 µg/mL. MIC: Minimum inhibitory concentration.

Minimum inhibitory concentration phenotype (µg/mL)	VanA	VanC1
0.125	0	0
0.25	0	0
0.5	1	0
1	5	0
2	3	0
4	1	0
8	0	0
16	0	0
32	6	4
64	21	0
128	17	0
>128	27	0

Among the 74 patients with VRE, isolates were most commonly found in individuals with comorbid conditions such as type 2 diabetes mellitus, hypertension, and other immunocompromised states. The median length of hospital stay was 13.5 days, with an interquartile range (IQR) of 14 days (Q1 = 7 days, Q3 = 21 days). Prior exposure to antibiotics was noted in 14/74 patients (18.9%) with VRE isolates. Prolonged use of invasive devices, such as urinary (Foley) catheters, Ryle's tubes, peripheral intravenous lines, central venous lines, arterial lines, and hemodialysis catheters, was also commonly noted. The median indwelling device duration was 6 days, with an IQR of 4-9 days. Of the 266 *Enterococcus *isolates, 25 (9.4%) were resistant to linezolid, and 13 of these were also resistant to vancomycin. Together, these findings suggest that VRE infections are closely linked to healthcare-associated risk exposures.

Statistical analysis

A statistically significant association was observed between VRE isolates and ICU admission (*p = 0.0013), with VRE isolates being more common among ICU patients compared to the ward patients. Statistical analysis interpretation is depicted in Table 6.

**Table 6:** Association of vancomycin resistance with admission wards and intensive care units among Enterococcus isolates *p-value < 0.05 = statistically significant. χ²: Chi-square value; df: Degrees of freedom; VRE: Vancomycin-resistant *Enterococcus*; VSE: Vancomycin-susceptible *Enterococcus*.

Isolate	Ward	ICU	Total	χ²	df	P-value*
VRE	48	26	74	10.34	df = 1	0.0013
VSE	161	31	192			
Total	209	57	266			

## Discussion

VRE is recognized as an important nosocomial pathogen because of the therapeutic challenges it poses and its capacity for dissemination within healthcare settings [4]. In the present study, 266 clinical *Enterococcus* isolates were analyzed to determine both the phenotypic and molecular basis of vancomycin resistance.

In this study, eight species of* Enterococcus* were identified from 266 isolates, with *Enterococcus faecalis* (48.1%) being the most common, followed by *E. faecium* (44.0%). A similar pattern has been observed ​​​​​in other studies, where *E. faecalis* tends to be more frequent than other species. However, our findings suggest an increasing proportion of *E. faecium* in the clinical setting [16,17].

In our study, vancomycin resistance was found in 27.8% (74/266) of the isolates. This proportion is considerably higher than that reported in earlier Indian studies, for example, 9.4% by Kaarthiga et al. [18], 14.7% by Sivaradjy et al. from South India [19], and 7.36% in a recent tertiary care study from North India [20]. It also exceeds the pooled Indian prevalence of 12.4% reported in a recent systematic review and meta-analysis [21]. Among the 74 VRE isolates, most were from urine (n = 34), followed by exudates (n = 32) and blood (n = 8). The majority of genotypically resistant isolates were from urine (40/84, 47.6%), followed by exudates (36/84, 42.9%) and blood (8/84, 9.5%). Of the VRE isolates, 49 were from male patients and 25 from female patients.

The species distribution in this study revealed a clear predominance of *E. faecium* among VRE isolates (57/74; 77.0%), with fewer isolates of* E. faecalis* (12.1%), *E. gallinarum* (9.5%), and *E. casseliflavus* (1.4%). This finding highlights the strong association of vancomycin resistance with *E. faecium*. For instance, a systematic review carried out in India (2000-2022) showed a predominance of *E. faecium* (58.15%) compared with *E. faecalis* (36.41%) among VRE isolates [21]. In addition, other clinical studies consistently show higher resistance rates in *E. faecium*, reinforcing its importance in vancomycin resistance [22].

The *vanA* genotype was the predominant resistance determinant identified among VRE isolates in the present study, with 70 (94.5%) isolates carrying *vanA* and only 3 (4%) carrying *vanC1*, while *vanB *was not detected in any isolate. This highlights the importance of *vanA* as the leading determinant of vancomycin resistance in clinical *Enterococci *isolates​​​​​. Notably, one isolate demonstrated the coexistence of both *vanA *and *vanC1*, suggesting the presence of both acquired and intrinsic resistance mechanisms within the same isolate. v*anC1*, typically found in intrinsically resistant species such as *E. gallinarum,* was detected only in a small proportion of isolates in this study [5].

These findings are consistent with prior reports showing a predominance of *vanA *and the absence or low prevalence of *vanB *in clinical isolates. For example, a similar pattern was observed by Moosavian et al. [23], with the presence of *vanA *and the absence of *vanB* in VRE isolates. Moreover, a global genomic analysis of *E. faecium* showed that the majority of *van *genes carrying isolates carried *vanA *(40.9%), with only a few isolates harboring *vanB *(5.8%) [24]. In addition, earlier studies have shown that intrinsic resistance genes such as *vanC1 *and *vanC2/3 *may be present in VRE isolates, occasionally coexisting with *vanA,* supporting the coexistence observed in our study [5].

In contrast to our findings, Karki et al. [25] reported* vanB* as the predominant resistance gene among VRE isolates and did not detect *vanA*. This variation may be due to geographic differences in resistance patterns. Furthermore, similar to the findings of Das et al. [15], none of our isolates carried *vanC2/3*, *vanD*, *vanE*, or* vanG *genes.

Compared with the earlier study conducted at our institution in 2016 [26], the present study found a higher prevalence of VRE, which increased from 12.9% [26] to 27.5%. The earlier study detected both *vanA *and *vanB *genes, with *vanB *being identified as more common. However, most isolates carrying *vanB* showed vancomycin MIC values within the susceptible range. In the present study, *vanA* was the predominant gene associated with vancomycin resistance. Only a small number of isolates carried *vanC1*, and *vanB *was not detected. These findings suggest that the pattern of vancomycin resistance among *Enterococcus* isolates in our institution has changed over time, with *vanA *now appearing to be the major mechanism of resistance.

Notably, out of 84/266 PCR-positive isolates, 10 were *vanA-*positive but were phenotypically susceptible to vancomycin. These findings were reconfirmed by repeat antimicrobial susceptibility testing and repeat PCR, which confirmed the presence of the* vanA* gene. This may suggest incomplete expression of the *vanA* gene, as previously reported by Kohler et al. [27] and Jordan et al. [28].

The control of VRE in healthcare settings primarily depends on four key measures, including periodic screening of high-risk patients, strict adherence to hand hygiene protocols, appropriate use of gloves and gowns along with cohorting of infected or colonized patients, and proper terminal cleaning of hospital rooms previously occupied by VRE-positive patients [29]. In addition, other approaches, such as antimicrobial stewardship to reduce unnecessary antibiotic use, decolonization strategies, and staff education programs, may provide further benefits in certain situations [30].

Study limitations

This study was carried out in a single tertiary care center, which may limit the generalizability of the findings. Reference strains carrying *vanC2/3*, *vanD*, *vanE*, and *vanG* resistance genes could not be obtained for inclusion as positive controls in the PCR. Additionally, due to resource constraints, sequencing of all PCR-positive isolates was not feasible. The 10 *vanA-*positive isolates showing phenotypic susceptibility were confirmed by repeat MIC-based antimicrobial susceptibility testing and repeat PCR to verify the presence of the *vanA *gene. However, alternative confirmatory methods such as E-test or broth microdilution were not performed, and further evaluation by transcriptional analysis or sequencing to assess *vanA *gene expression was not carried out due to resource constraints.

## Conclusions

This study highlights the increasing prevalence of VRE and the dominance of the *vanA* genotype, while *vanC1* was detected in a small proportion of isolates. The emergence of linezolid resistance is concerning, as it further limits treatment options. This study demonstrated co-resistance to vancomycin and linezolid in 13 isolates. Detection of* vanA* in phenotypically susceptible isolates highlights the importance of molecular confirmation in addition to susceptibility testing. These findings collectively emphasize the significance of continuous surveillance and robust antimicrobial stewardship in monitoring evolving resistance trends in *Enterococci.*
